# Physical activity motivations and psychological well-being among university students: a canonical correlation analysis

**DOI:** 10.3389/fpubh.2024.1442632

**Published:** 2024-10-08

**Authors:** Tao Zhong

**Affiliations:** College of Sport and Health, Henan Normal University, Xinxiang, China

**Keywords:** physical activity, self-determination theory, motivation, psychological well-being, canonical correlations, university students

## Abstract

With increasing concern about mental health issues and active lifestyles among university students, understanding the interplay between different physical activity motivations and various dimensions of psychological well-being is important. The present study aims to explore the canonical relationship between physical activity motivations based on self-determination theory and psychological well-being according to Ryff’s model in university students. Nine hundred and sixty-six Chinese university students participated in this study. A canonical correlation analysis was conducted using six variables of motivations as predictors of six variables of psychological well-being. The canonical correlation analysis yielded two canonical functions. The first canonical function, which was primary, indicated that intrinsic motivation, integrated regulation, identified regulation, and introjected regulation contributed the most to psychological well-being. The second canonical function indicated that a decrease in external motivation and amotivation accounted for an increase in personal growth. This study underscores the importance of elucidating the underlying motivations driving physical activity behaviors in order to enhance psychological well-being in this population.

## 1 Introduction

University students are a population at high risk for mental health issues (1). They are transitioning from adolescence to adulthood, which is characterized by significant changes and challenges, including increased responsibilities, stressors, and the need to navigate various life domains (2). Hence, university students may experience higher levels of stress, anxiety, and depression during this period. The results of the WHO World Mental Health Surveys International College Student Project indicated that the 12-month prevalence rates of major depressive disorder and generalized anxiety disorder were 18.5 and 16.7% (3). In a meta-analytic study, the pooled prevalence of depression was found to be 25%, while the pooled prevalence of suicide-related outcomes was determined to be 14% (1). In an investigation conducted during the Coronavirus Disease 2019 (COVID-19) pandemic among university students, the prevalence of stress, depression, and generalized anxiety symptoms was found to be even higher in the sampled population, at 61.3, 40.3, and 30%, respectively (4).

To support psychological well-being of university students, understanding relevant correlates is important. Extant literature has shown that an array of factors could affect their well-being. For instance, research has found that higher level of neuroticism, as a personality trait, was consistently linked with a lower level of psychological well-being, whereas traits of extraversion and consciousness demonstrated a positive connection with psychological well-being (5). Besides, individuals’ internal and external resource factors, including self-efficacy, resilience, mindfulness, hopeful and optimistic thinking, and social support had a positive effect on psychological well-being (6, 7).

Moreover, health-related behaviors, such as physical activity, have been identified as critical for impacting the psychological well-being of university students (8). While physical activity is known to play a crucial role in psychological well-being, the extent of physical activity engagement among university students varies. Research indicates that while some students adhere to recommended physical activity levels, others engage in minimal or no physical activity (9, 10). Moreover, although physical activity behavior itself is important, it is also critical to consider the underlying motivations driving physical activity behavior, and analyze how these motivations may relate to psychological well-being among university students. In this regard, self-determination theory, as an important contemporary motivation theory (11), could be a useful framework for exploring motivations for physical activity.

Self-determination theory proposes that human behavior is driven by various types of motivations. Along a continuum of internalization and from the most internalized to the least, they are intrinsic motivation, integrated regulation, identified regulation, introjected regulation, external regulation and amotivation (12). Intrinsic motivation, as the most autonomous form of motivation, is driven by an interest or enjoyment in the activity itself. Integrated regulation is characterized by a sense of congruence between a behavior and self. Identified regulation is the next level down on the motivation continuum, and it is characterized by a sense of personal importance and relevance. Introjected regulation represents motivation out of internalized pressures, such as guilt or shame; or to improve the ego, feelings of value, or pride. External regulation drives behavior out of the purpose of obtaining rewards or avoiding punishment (13). Amotivation, as the least self-determined pole of motivation continuum, is the absence of motivation. Individuals who are amotivated may not have any interest or enjoyment in an activity, and may not see any value or purpose in it (14).

In the context of physical activity, some empirical research has been conducted on how a single type of motivation could lead to different outcomes (15). For instance, in regression models, intrinsic motivation has been consistently shown to be linked to physical activity enjoyment, long-term behavioral engagement, positive emotional experiences such as excitement, and reduced stress, anxiety, and depression (16). While such an approach can be useful, it cannot clarify how different motivations function together in effecting outcomes. In this regard, the use of canonical correlation analysis can extend this idea by investigating the relationship between two groups of variables and capturing higher-dimensional relationships between group variables (17).

Another point to note is that when testing the associations, well-being is often assessed as a single construct, such as satisfaction with life or positive affect (18). In this regard, Ryff has proposed a comprehensive model to capture the rich and nuanced meanings of well-being (19). As a widely used theory in positive psychology, Ryff’s psychological well-being framework comprises six dimensions: self-acceptance, personal growth, purpose in life, positive relations with others, environmental mastery, and autonomy. Self-acceptance indicates the acceptance and appreciation of oneself, including one’s strengths and imperfections. Personal growth is the pursuit of self-actualization and the development of new skills, knowledge, and perspectives. Purpose in life is the sense that one’s life has direction and meaning, and that one’s actions are guided by a sense of purpose. Positive relations with others relate to the quality of relationships with others. Environmental mastery concerns being effective in one’s daily life and being able to adapt to changes. Finally, autonomy refers to the ability to make choices and have control over one’s life. These dimensions are considered to collectively contribute to an individual’s overall psychological well-being (20). As yet, however, it remains unclear how different motivations could be connected with different aspects of psychological well-being.

Therefore, the present study aims to investigate the relationship between physical activity motivations and psychological well-being among university students using canonical correlation analysis. By elucidating how different motivations for physical activity may be related to various aspects of psychological well-being, this study seeks to provide valuable insights for promoting well-being and mental health in university students.

## 2 Method

### 2.1 Participants

The study included 966 Chinese undergraduate students, with 566 women (58.6%) and 400 men (41.4%), from a large university in China. The participants were between 18 and 23 years old, with an average age of 19.16 ± 1.11 years. The students came from various academic programs, covering areas of natural sciences, engineering, social sciences, and humanities. Although the participants’ physical activity levels were not directly assessed, they had some physical activity experiences, as they attended physical education classes and underwent physical fitness examinations every semester. The characteristics of the study participants, including age, gender and academic backgrounds are presented in Table 1.

**Table 1:** Characteristics of the university student participants (*N* = 966).

	*N*	%
Age		
18	303	31.37
19	372	38.51
20	180	18.63
21	57	5.90
22+	54	5.59
Gender		
Male	400	41.41
Female	566	58.59
Academic backgrounds		
Natural science and engineering	408	42.24
Humanities and social science	240	24.84
Sport and health science	294	30.43
Missing values	24	2.48

### 2.2 Procedure

An online questionnaire was created on a professional survey platform Wen Juan Xing, which is broadly used in China. Subsequently, colleagues of the author’s affiliation were contacted for permission to administer the survey to students. An invitation pamphlet was sent to potential participants, including information about the study and a QR code that allowed them to access the survey. Participants were informed that their participation was voluntary and anonymous, and their response would be kept confidential. Also, they were assured that they could withdraw at any time. By clicking on an opt-in button and completing the survey, participants indicated their consent to participate. Besides, to ensure data integrity, the author used a setting in Wen Juan Xing that allowed only one device (such as a mobile phone or a computer) to submit a response once. However, there was still a possibility that a participant might use more than one device to respond to the survey multiple times. Therefore, the author conducted a quality check on the data, which did not find any responses that could imply they came from the same person. The study was approved by the ethics committee at the author’s institution and was conducted in accordance with the Declaration of Helsinki and its later amendments.

### 2.3 Measures

Physical activity motivation. It was assessed with the Sport Motivation Scale-6 (SMS-6) (21). The SMS-6 is a self-report measure designed to assess six types of motivation in sport based on self-determination theory. The scale consists of 24 items, with four items for each subscale assessing a specific type of motivation. Items are rated on a 7-point Likert scale, ranging from 1 (do not correspond at all) to 7 (correspond exactly). A sample item reads: “For the excitement I feel when I am really involved in the activity.” The scores for each subscale are calculated by summing and averaging the ratings for the corresponding items. The external validity of the scale was supported by its correlations with theoretically related constructs. For example, research has investigated the scale’s correlation with psychological flow. Flow is a pleasurable experience during physical activity participation, and it is a positive mental state characterized by complete immersion, energized focus, full involvement, and enjoyment (22). It is theorized that individuals who are autonomously motivated should be more likely to experience flow due to their stronger sense of personal connection with the activity (23, 24). Research has demonstrated that the autonomous forms of physical activity motivation, such as intrinsic motivation, were significantly positively correlated with psychological flow, whereas external regulation and amotivation showed non-significant correlations or negative correlations with psychological flow (21), which supports the external validity of the scale. Regarding its internal reliability, in the current study, the values of internal consistency reliability (Cronbach’s *α*) were 0.890 for intrinsic motivation, 0.914 for integrated regulation, 862 for identified regulation, 827 for introjected regulation, 760 for external regulation, and 0.812 for amotivation.

Psychological well-being. It was assessed with Ryff’s 42-item Psychological Well-being Scale (25). The scale measures six well-being dimensions, with each assessed with seven positively or negatively worded items. The items are anchored on a 7-point Likert type scale, ranging from 1 (strongly agree) to 7 (strongly disagree). In line with previous research (26), only positively worded items were used to calculate a score for each well-being dimension, and items were recoded so that a higher point indicates a higher level of well-being. A sample item reads: “Most people see me as loving and affectionate.” In terms of the external validity of the scale, past research has included theoretically linked measures of happiness, life satisfaction and depression to test its external validity. As expected, positive associations were found between measures of happiness and life satisfaction and psychological well-being dimensions. Conversely, the severity of depressive symptoms was negatively associated with psychological well-being dimensions (19). Additionally, a negative association between the psychological well-being scale and a summary measure of psychological distress (the General Health Questionnaire) has been reported, which further substantiates the external validity of the scale (27). Regarding the internal reliability of the scale, in the current study, the values of internal consistency reliability (Cronbach’s *α*) were 0.841 for self-acceptance, 0.876 for personal growth, 0.857 for purpose in life, 0.876 for positive relations with others, 0.843 for environmental mastery and 0.825 for autonomy.

### 2.4 Data analysis

Prior to main analysis, descriptive analysis (e.g., mean and standard deviation) and bivariate correlation were employed. Successively, canonical correlation analysis was conducted to examine the relationship between physical activity motivations and psychological well-being. It is a multivariate statistical technique that evaluates the relationships between two sets of variables, with one set serving as the predictor and the other as the criterion (28). In this study, physical activity motivations were the predictor set and dimensions of psychological well-being were the criterion set. The canonical correlation analysis identified the canonical functions that best predicted the relationships between the predictor and criterion sets (29). The magnitude of the canonical correlations and their associated *p*-values provided an indication of the strength and significance of the relationship between physical activity motivations and psychological well-being. The canonical loadings, which represented the correlation between the original variables and their canonical variates, were used to determine the relative importance of each predictor variable in defining the canonical function (30). In line with previous research recommendations, only canonical functions with coefficients exceeding |0.30| were considered meaningful and interpreted in this study (31). The analyses were performed in SPSS 26.0.

## 3 Results

The descriptive statistics and bivariate correlation results are displayed in Table 2. In terms of the bivariate correlations between physical activity motivations and psychological well-being, intrinsic motivation, integrated regulation, identified regulation and introjected regulation exhibited a comparable strength of positive correlation with dimensions of psychological well-being. External motivation was also found to be positively correlated with dimensions of psychological well-being, albeit to a lesser extent. Amotivation was not significantly correlated with psychological well-being, with the exception of a weak and negative correlation with personal growth.

**Table 2:** Descriptive statistics and bivariate correlation of physical activity motivations and psychological well-being variables.

Variables	*M*	SD	Skew.	Kurt.	*α*	1	2	3	4	5	6	7	8	9	10	11
1. INTRI	5.264	1.290	−0.445	−0.292	0.890	–										
2. INTE	5.153	1.349	−0.377	−0.413	0.914	0.907^**^	–									
3. IDEN	5.139	1.282	−0.335	−0.283	0.862	0.903^**^	0.912^**^	–								
4. INTRO	4.897	1.285	−0.165	−0.289	0.827	0.816^**^	0.853^**^	0.861^**^	–							
5. EXTER	4.296	1.348	0.184	−0.366	0.760	0.691^**^	0.733^**^	0.762^**^	0.790^**^	–						
6. AM	3.456	1.460	0.304	−0.267	0.812	−0.001	0.016	0.063	0.178^**^	0.355^**^	–					
7. SA	4.547	1.220	−0.087	0.240	0.841	0.380^**^	0.384^**^	0.392^**^	0.362^**^	0.320^**^	0.032	–				
8. PG	5.087	1.334	−0.680	0.547	0.876	0.378^**^	0.353^**^	0.361^**^	0.318^**^	0.166^**^	−0.065^*^	0.750^**^	–			
9. PL	4.744	1.351	−0.276	0.046	0.857	0.419^**^	0.430^**^	0.428^**^	0.398^**^	0.290^**^	−0.035	0.786^**^	0.817^**^	–		
10. PRO	4.797	1.277	−0.349	0.318	0.876	0.412^**^	0.401^**^	0.415^**^	0.373^**^	0.280^**^	−0.023	0.823^**^	0.843^**^	0.803^**^	–	
11. EM	4.732	1.287	−0.282	0.102	0.843	0.418^**^	0.423^**^	0.425^**^	0.397^**^	0.313^**^	−0.006	0.866^**^	0.834^**^	0.838^**^	0.871^**^	–
12. AU	4.577	1.207	−0.129	0.372	0.825	0.387^**^	0.403^**^	0.382^**^	0.375^**^	0.287^**^	0.012	0.811^**^	0.797^**^	0.807^**^	0.795^**^	0.851^**^

M, mean; SD, standard deviation; Skew., skewness; Kurt., kurtosis; α, Cronbach’s alpha coefficient; INTRI, intrinsic motivation; IDEN, identified regulation; INTE, integrated regulation; INTRO, introjected regulation; EXTER, external regulation; AM, amotivation; SA, self-acceptance; PG, personal growth; PL, purpose in life; PRO, positive relations with others; EM, environmental mastery; AU, autonomy. ^*^p < 0.05, ^**^p < 0.01, ^***^p < 0.001.

In line with the number of study variables in the present study, the number of canonical functions that could be generated in the canonical correlation analysis was six. Their canonical function coefficients were 0.469, 0.339, 0.160, 0.093, 0.027, 0.005, and their Wilks’s Lambda values were 0.667, 0.855, 0.965, 0.991, 0.999 and 1.000, respectively. The canonical function coefficients of the last four functions were markedly inferior to the acceptable value of |0.30|, and they accounted for only minimal amounts of the variance between physical activity motivations and psychological well-being (2.560, 0.865, 0.073, and 0.003%). Hence, they were not considered further, and only the first two canonical functions were deemed to represent interpretable and meaningful canonical associations between physical activity motivations and psychological well-being. Specifically, the first canonical function had *R_c1_* = 0.469, Wilks’s Lambda = 0.667, *F*(36, 4192.068) = 11.257, *p* < 0.001, and the second canonical function had *R_c2_* = 0.339, Wilks’s Lambda = 0.855, *F*(25, 3549.169) = 6.132, *p* < 0.001. Table 3 presents the canonical loadings for both canonical functions retained from the canonical correlation analysis. The first canonical function, which was primary, had an eigenvalue of 0.282 and could explain 21.996% of the variance between the canonical variates. Besides, it could account for 67.395% of the variance in physical activity motivations and 79.521% of the variance in psychological well-being. An inspection of the loadings indicated that strong contributions to the first canonical function were made by scores on intrinsic motivation, integrated regulation, identified regulation and introjected regulation. External regulation had a lower loading and amotivation had a negligible loading on the first canonical function. In terms of the contribution of set 2 variables (i.e., psychological well-being), strong contributions could be observed from self-acceptance, personal growth, purpose in life, positive relations with others, environmental mastery, and autonomy, with all canonical loadings larger than |0.800|. Purpose in life demonstrated the strongest canonical loading. Thus, the results showed that increased intrinsic motivation, integrated regulation, identified regulation and introjected regulation were positively associated with self-acceptance, personal growth, purpose in life, positive relations with others, environmental mastery, and autonomy. While external regulation was also positively correlated with psychological well-being, the strength of this correlation was notably smaller.

**Table 3:** Canonical loadings of physical activity motivations and psychological well-being dimensions.

	Canonical loadings	
Variables	Function 1	Function 2
Types of motivation		
Intrinsic motivation	−0.942	0.037
Integrated regulation	−0.964	−0.142
Identified regulation	−0.963	−0.085
Introjected regulation	−0.901	−0.200
External regulation	−0.698	−0.668
Amotivation	0.041	−0.399
Psychological well-being		
Self-acceptance	−0.840	−0.149
Personal growth	−0.830	0.485
Purpose in life	−0.958	0.084
Positive relations with others	−0.909	0.122
Environmental mastery	−0.937	−0.013
Autonomy	−0.869	−0.027

Both canonical functions were significant at *p* < 0.001.

The second canonical correlation function, which was secondary, had an eigenvalue of 0.130 and could explain 11.492% of the variance between the canonical variates. Additionally, it could account for 11.227% of the variance in physical activity motivations and 4.673% of the variance in psychological well-being. An inspection of its canonical loadings indicated that the main contributors in the first set of variables (motivations) to this canonical function were external motivation and amotivation, with negative canonical loadings of an absolute value larger than |0.30|; among the second set of variables (psychological well-being), personal growth emerged as the most important contributor, with a canonical loading of 0.485; in the meantime, the rest of the variables had relatively smaller canonical loadings (<|0.15|). Thus, the results showed that with the decrease of external regulation and amotivation, personal growth would increase.

## 4 Discussion

The present study aims to investigate the relationship between physical activity motivations and psychological well-being among university students. The results of the canonical correlation analysis revealed two significant canonical functions, which accounted for a moderate degree of association between the two sets of variables. The first canonical function, which was primary, indicated that intrinsic motivation, integrated regulation, identified regulation, and introjected regulation were positively associated with psychological well-being, as measured by Ryff’s comprehensive psychological well-being model. The findings of this study support the self-determination theory (32), which proposes that more internalized and self-determined motivations, such as intrinsic motivation, were positively correlated with psychological well-being (33).

Specifically, intrinsic motivation could be positively linked to self-acceptance by fostering a positive self-view through engaging in enjoyable and self-affirming activities (34). University students who find joy in their physical activity may thus be more likely to have a positive self-image and greater satisfaction with themselves. Regarding its association with personal growth, research has suggested that engaging in activities that provide personal satisfaction and challenge can lead to personal growth (35). Thus, university students who are intrinsically motivated to participate in physical activity could be more likely to experience enhanced self-development. In addition, intrinsic motivation may be positively linked to the psychological well-being dimension of purpose in life, as demonstrated in the study. This could be explained in that intrinsic motivation which aligns with personal interests and values could cultivate a more profound sense of purpose and fulfillment, which in turn contributes to a sense of purpose and meaning (36, 37).

While intrinsic motivation often focuses on personal enjoyment, it may also lead to improved social interactions when activities are pursued in group settings or involve social elements (38). Engaging in enjoyable physical activity with peers could thus potentially strengthen relationships and enhance social support. Intrinsic motivation may also be positively associated with the psychological well-being dimension of environmental mastery because by pursuing activities that are inherently enjoyable, university students would be able to develop skills and competencies that enhance their ability to manage their environment effectively; and further, mastery in activities could translate to greater confidence in handling other aspects of life (39). Finally, in terms of the connection between intrinsic motivation and the psychological well-being dimension of autonomy, it could be viewed that when university students choose physical activity that align with their interests and values, they may be more likely to experience a sense of autonomy and control which contributes to overall well-being (37, 40).

The above nuanced findings about how more self-determined motivation such as intrinsic motivation impacts on different dimensions of psychological well-being could benefit the university student population by providing valuable insights into targeted educational efforts. Based on self-determination theory, creating physical activity opportunities that foster basic psychological needs fulfillment including autonomy, competence and relatedness are important for physical activity behavioral internalization and motivation internalization (32, 41). Therefore, universities should provide opportunities for enjoyable and fulfilling physical activity that can support university students’ psychological well-being.

It should be noted that there is some controversy over introjected regulation. Specifically, it has demonstrated both a positive and a non-significant relation with adaptive outcomes among studies undertaken in Western countries (15). In the present study which involved Chinese participants, its positive relation with psychological well-being was revealed. This result could also be observed in empirical research conducted among Chinese individuals (42). In Chinese culture, collectivism is emphasized over individualism, which can lead to a stronger emphasis on social harmony and group cohesion (43). This can result in individuals being more likely to engage in introjected regulation, as they may feel a strong sense of responsibility to maintain social relationships and avoid conflict (44). Therefore, culture may have played a role in endorsing and reinforcing this motivation among the Chinese participants, which could lead to a positive association with well-being.

In addition, external regulation had a lower loading, while amotivation had a negligible loading on the first canonical function; this suggests that while external regulation may be positively correlated with psychological well-being, its strength was smaller. For amotivation, it was not significantly associated with psychological well-being. The second canonical function, which was secondary, indicated that external motivation, amotivation in the first set, and personal growth in the second set, were the main contributors. The findings suggest that decreased external regulation and amotivation were associated with increased personal growth. Taken together, external regulation had a positive yet weaker association with psychological well-being in the first canonical function, and a negative association with personal growth in the second function. Therefore, as a less internalized and self-determined type of motivation, at least it should not be advocated in regulating physical activity participation (35).

Besides, amotivation was found to play an insignificant or negative role in psychological well-being. Amotivation is a lack of intention, motivation or interest, which is often accompanied by a sense of disengagement, boredom, and disinterest. Therefore, it could lead to decreased psychological well-being (45). Specifically, amotivation may undermine the psychological well-being dimension of self-acceptance (46). This may be because university students who experience amotivation often feel that their efforts are futile, and such perceptions of ineffectiveness in physical activity may negatively impact self-acceptance as individuals struggle to embrace themselves positively. The identified negative association between amotivation and personal growth suggests that amotivation may diminish the opportunities for personal growth and skill development thereby limiting the potential for self-improvement and personal development (36).

Additionally, amotivation could be linked to a diminished sense of purpose and meaning, as university students oriented with amotivation may lack direction or goals related to physical activity, which can affect overall life satisfaction. Amotivation might also lead to withdrawal from social activities or interactions, potentially weakening social connections and support networks. Furthermore, individuals with amotivation might struggle with managing their environment effectively due to a lack of engagement and confidence in their abilities (47). Finally, amotivation is associated with a perceived lack of control or choice in engaging in physical activity, which could result in a reduced sense of autonomy and self-direction. Given the negative connection of amotivation with various dimensions of psychological well-being as discussed above, it is important to address barriers to motivation and enhance university students’ internalized motivation and behavioral regulation in physical activity, as this may help protect their psychological well-being (48).

Findings of the study emphasize the need for physical activity programs and interventions to consider the quality of motivation when targeting university students. Fostering more internalized motivations, such as intrinsic motivation, integrated regulation, and identified regulation, may be an effective way to enhance psychological well-being, along with promoting long-term engagement in physical activity as shown in earlier research (15). By cultivating a sense of enjoyment and personal interest in physical activity, as well as connecting it to one’s values and identity, interventions can potentially enhance psychological well-being and promote long-term engagement in physical activity. This is particularly relevant for university students, who are at a critical stage of identity formation and may be more receptive to interventions that align with their personal values and interests (49).

Notwithstanding the findings, limitations associated with this study need to be acknowledged. The study’s cross-sectional design limits the ability to establish causality between physical activity motivations and psychological well-being. Longitudinal research is needed to examine the directionality and long-term effects of these relationships in this population. Additionally, the generalizability of the findings may be limited by the use of a convenience sample, and future research should aim to include more diverse and representative samples.

In conclusion, this study provides valuable insights into the connection between physical activity motivations based on self-determination theory and psychological well-being as per Ryff’s psychological well-being model among university students. The significant relationship established through canonical correlation analysis highlights the importance of motivational quality in shaping psychological well-being in the physical activity context. As a result, it is crucial to incorporate these findings into the design of effective physical activity interventions for university students to enhance their psychological well-being.

## Data Availability

The raw data supporting the conclusions of this article will be made available by the author, without undue reservation.
